# Stress and eating: a dual role for bombesin-like peptides

**DOI:** 10.3389/fnins.2013.00193

**Published:** 2013-10-25

**Authors:** Z. Merali, S. Graitson, J. C. MacKay, P. Kent

**Affiliations:** ^1^Department of Psychology, University of OttawaOttawa, ON, Canada; ^2^Department of Cellular and Molecular Medicine, University of OttawaOttawa, ON, Canada; ^3^University of Ottawa Institute of Mental Health ResearchOttawa, ON, Canada; ^4^Department of Biomedical Sciences, University of OttawaOttawa, ON, Canada

**Keywords:** gastrin-releasing peptide, neuromedin B, stress, reward, obesity, anorexia

## Abstract

The current obesity “epidemic” in the developed world is a major health concern; over half of adult Canadians are now classified as overweight or obese. Although the reasons for high obesity rates remain unknown, an important factor appears to be the role stressors play in overconsumption of food and weight gain. In this context, increased stressor exposure and/or perceived stress may influence eating behavior and food choices. Stress-induced anorexia is often noted in rats exposed to chronic stress (e.g., repeated restraint) and access to standard Chow diet; associated reduced consumption and weight loss. However, if a similar stressor exposure takes place in the presence of palatable, calorie dense food, rats often consume an increase proportion of palatable food relative to Chow, leading to weight gain and obesity. In humans, a similar desire to eat palatable or “comfort” foods has been noted under stressful situations; it is thought that this response may potentially be attributable to stress-buffering properties and/or through activation of reward pathways. The complex interplay between stress-induced anorexia and stress-induced obesity is discussed in terms of the overlapping circuitry and neurochemicals that mediate feeding, stress and reward pathways. In particular, this paper draws attention to the bombesin family of peptides (BBs) initially shown to regulate food intake and subsequently shown to mediate stress response as well. Evidence is presented to support the hypothesis that BBs may be involved in stress-induced anorexia under certain conditions, but that the same peptides could also be involved in stress-induced obesity. This hypothesis is based on the unique distribution of BBs in key cortico-limbic brain regions involved in food regulation, reward, incentive salience and motivationally driven behavior.

## Introduction

The worldwide prevalence of obesity has doubled since 1980 and we have entered what is being called a “tsunami of obesity.” According to the 2008 Statistics Canada report, 61% of adult Canadians were overweight or obese, contributing to and/or exacerbating outcomes of various health conditions including cardiovascular disease, type II diabetes, sleep apnea, as well as many psycho-social disorders (Stein and Colditz, 2004).

While the causes of the obesity epidemic are complex, stress has been identified as an important factor. Increased rates of obesity have been accompanied by a concomitant rise in perceived stress in North America. In humans, greater reported stress is associated with greater desire to eat, including binge eating (Warne, 2009). Further, high levels of perceived stress correlate with weight gain and obesity, as women who self-identify as high-stress responders to a laboratory stressor, have significantly greater BMI and sagittal diameters, than low-stress responders (Tomiyama et al., 2011). Similarly, students who self-identify as stress-eaters have higher levels of stress hormones like cortisol, during stressful periods, such that the elevated cortisol may be associated with their increased desire to eat (Epel et al., 2004).

While increased indices of stress in society are a tempting explanation for the obesity epidemic, it may be deceptively simple, as stress appears to affect feeding in a bidirectional manner. In humans, stress causes increased food intake in one subset of the population and conversely causes decreased food intake in another; why some people lose weight and other gain weight is not yet well understood (Stone and Brownell, 1994; Epel et al., 2004). Stress-induced anorexia is also commonly seen in animal research, where rats fed a standard chow diet lose weight or decrease food intake in response to chronic stress (e.g., repeated restraint or variable stressors) (Martí et al., 1994; Harris et al., 1998; Pecoraro et al., 2004). Indeed stress severity can alter Chow intake, such that the greater the severity of the stressor, the greater the suppression of Chow intake (Torres and Nowson, 2007; Maniam and Morris, 2012). However, there is also evidence of rodents that alternatively increase food consumption or gain weight in response to chronic stress, in particular, repeated social defeat (Foster et al., 2006; Tamashiro et al., 2007a,b). It bears noting that the stressor paradigms used by researchers, vary widely in terms of duration, intensity and nature (i.e., systemic, neurogenic, psychosocial etc.), making it exceedingly challenging to categorize the varied feeding responses.

Beyond the nature of the stressor itself, the type of food available appears to vary greatly, potentially contributing to the discrepant findings ranging from stress-induced anorexia to stress-induced obesity. In rats, while Chow consumption often decreases following repeated stress, consumption of tasty, calorie dense “palatable” food (typically high fat/high sugar content) remains unaffected (Ortolani et al., 2011) and the proportion of palatable food eaten relative to standard Chow can increase (Pecoraro et al., 2004). Additionally, while stress often induces weight loss in rats fed standard lab Chow, this weight loss can be reversed if given access to a palatable food diet (Harris et al., 1998; Pecoraro et al., 2004; Ortolani et al., 2011). Further, our lab has shown that palatable food consumption exacerbated the effects of a stressed rat's ability to handle a glucose load challenge and led to increased accumulation of visceral fat (MacKay et al., 2011), indicating that the combination of stressor exposure and access to palatable food may predispose individuals to developing metabolic syndrome and/or obesity later in life.

The interactions between feeding regulation and stress must be complex to produce such varied phenotypes; indeed, feeding regulation under non-stress conditions involves many interacting signals, and the mechanism(s) becomes increasingly complex when stress is introduced (Torres and Nowson, 2007; Maniam and Morris, 2012). It is not surprising, then, that many of the regulatory systems and circuitry that govern feeding are sensitive to stress. Several neural signals, neuropeptides in particular, serve dual roles as regulators of both feeding and stress response, and are thus well-positioned to mediate stress-induced changes in feeding behavior. These peptides include (but are not limited to) corticotropin-releasing factor (CRF), leptin, ghrelin, orexin, neuropeptide Y, melanocortin and cholecystokinin (Crawley and Corwin, 1994; Dallman et al., 1995; Hanson and Dallman, 1995; Merali et al., 1998; Koob and Heinrichs, 1999; Ahima and Flier, 2000; Vergoni and Bertolini, 2000; Dhillo et al., 2002; Ueta et al., 2003; Spinazzi et al., 2006; Stevanović et al., 2007; Kirsz and Zieba, 2011; Barson et al., 2013).

Another family of peptides similarly implicated in both feeding and stress is the bombesin-like peptides (which will henceforth be referred to as BBs). Bombesin, a 14 amino acid peptide first isolated from the skin of the frog *Bombina bombina* (Erspamer et al., 1970), originally generated intense interest because of its potent biological actions in mammals (Panula, 1986). Two mammalian bombesin homologs were subsequently discovered including gastrin-releasing peptide (GRP) and neuromedin B (NMB) (McDonald et al., 1979; Minamino et al., 1983, 1988). Appropriate receptors have also been identified (Minamino et al., 1988; Spindel et al., 1990; Battey and Wada, 1991; Jensen et al., 2008): whereas GRP has a greater affinity for BB_2_ receptors, NMB preferentially activates the BB_1_ receptor subtype (Spindel et al., 1990; Battey and Wada, 1991; Jensen et al., 2008), and the BB_3_ receptor is a structurally related orphan receptor whose endogenous ligand remains unidentified (Weber et al., 1998).

BBs have long been recognized for their satiety properties as they are able to shorten meal size and duration of all mammals tested [for reviews, see (Merali et al., 1999; Yamada et al., 2002)]. Exogenous BB administration also activates the hypothalamic-pituitary-adrenal (HPA) axis and endogenous BBs are released during stressor exposure suggesting a role in mediation and/or modulation of the stress response (Merali et al., 2002). These facts, which will be expanded upon below, provide the framework for our first contention; that BBs play a role in stress-induced anorexia. However, beyond this more obvious role, we also contend that when stressor exposure is combined with a palatable food diet, the satiety effects of BB are superseded by extra-hypothalamic (cortico-limbic) BBs that *promote* obesity. This contention is based on the following which will be outlined in detail below: BBs (1) are released in response to not only aversive events (stressor exposure), but appetitive (food reward) events as well (Merali et al., 1998); and (2) are specifically localized in key brain regions involved in both stress and reward circuits, where they influence motivationally driven behavior (Merali et al., 2004, 2011, 2013; Mountney et al., 2008).

## Palatable food impacts feeding response to stress

Palatable food may be distinguished from regular Chow because it is capable of activating neural reward circuitry. The powerful rewarding properties of food have been paralleled to those of drugs of abuse, and thus overeating has been compared to addiction (Dagher, 2009; Avena and Gold, 2011). Removal of a palatable diet can induce withdrawal-like behaviors (Cottone et al., 2009) and can cause rodents to endure aversive stimuli in order to regain access to palatable food (Pickering et al., 2009). Interestingly, stress is implicated in the reinstatement of not only substance abuse among abstinent drug users but also of failure among dieters (Adam and Epel, 2007). Stress and food reward both activate a broad array of neurocircuits involving several brain regions, including limbic [amygdala, nucleus accumbens (NAcc)] and cortical areas [anterior cingulate cortex (ACC)] (Lutter and Nestler, 2009; Dallman, 2010); circuits that often overlap.

It is noteworthy that the ability of palatable food to activate reward circuitry is associated with another phenomenon, whereby access to palatable food can mitigate or dampen the effects of stressors (Pecoraro et al., 2004; Dallman et al., 2005; Ulrich-Lai et al., 2010). Access to so-called “comfort food” appears to diminish the activation of the HPA axis in response to stress. This is reflected by attenuated release of adrenocorticotropic hormone (ACTH) and corticosterone following acute (restraint) stress (Kinzig et al., 2008; Foster et al., 2009; Christiansen et al., 2011a) and chronic stress (Pecoraro et al., 2004; Ulrich-Lai et al., 2007; Maniam and Morris, 2010b). In addition, consumption of palatable food has also been linked to improved emotional states, as reflected by reduced anxiety- and depressive-type behaviors (Maniam and Morris, 2010a,b; Ulrich-Lai et al., 2010). Consistent with these reports, our lab recently showed that resting and stressor-induced levels of corticosterone were attenuated in rats with access to the palatable (or comfort) foods, compared to controls that only had access to Rat Chow (or “mundane” food). In addition, episodic stressor exposure during the juvenile period is also associated with profound long-term anxiety and this effect is attenuated by access and consumption of comfort food (MacKay et al., 2011). The ability of comfort food to dampen the effects of stress appears to be linked to its hedonic value, as oral consumption of sucrose or non-caloric sweetener also provides stress-buffering effects, while intra-gastric gavage of sucrose does not (Ulrich-Lai et al., 2010).

The increasing levels of perceived societal stress accompanied by stress-dampening properties of comfort food may, in part, contribute to the obesity epidemic. It has been suggested that the stress relief provides negative reinforcement for the consumption of palatable food (Parylak et al., 2011). The learned association between comfort food and stress relief may result in the habitual consumption of comfort food in response to stress (Dallman, 2010), particularly given that stress promotes habitual behavior at the expense of goal-directed behaviors (Schwabe and Wolf, 2009).

## Feeding and stress: overlapping neural circuitry

The decision to eat or not to eat is regulated by two parallel and interacting systems, namely the homeostatic and the non-homeostatic systems (Kelley et al., 2005; Lutter and Nestler, 2009). The homeostatic system includes classic hypothalamic and brainstem pathways that govern energy balance in response to nutrient availability (Suzuki et al., 2010). The hypothalamus is a key region where many feeding circuits converge, (Benarroch, 2010; Maniam and Morris, 2012; Sinha and Jastreboff, 2013); this region is also known to participate in the mediation of the stress response. Stress activates the HPA axis, causing cascading release of CRF from the hypothalamus, ACTH from the anterior pituitary, and finally glucocorticoids (GCs) from the adrenals. It is noteworthy that these essential stress signals also have effects on feeding; in fact, central administration of CRF inhibits feeding (Arase et al., 1988), while central administration of GCs promotes feeding (Dallman et al., 2007). Within the hypothalamus, the arcuate nucleus contains two populations of neurons; one that stimulates feeding and one that inhibits feeding (Benarroch, 2010; Suzuki et al., 2010). These neurons project to the lateral hypothalamus, which communicates with reward circuitry (to be further discussed later), and the paraventricular nucleus of the hypothalamus (PVN) where CRF-producing neurons initiate the HPA axis cascade of stress response (Suzuki et al., 2010; Pandit et al., 2011). The hypothalamic nuclei are also responsive to several feeding signals, including insulin, leptin, and GCs (Dallman et al., 2006; Maniam and Morris, 2012; Sinha and Jastreboff, 2013). It is worthy of note that BBs, (particularly GRP) and their receptors as well as BB_3_ receptor mRNA are highly localized in the hypothalamus at key feeding sites including the PVN and arcuate nucleus (Battey and Wada, 1991; Ladenheim et al., 1992; Zhang et al., 2013).

The homeostatic pathways are then embedded in a much larger neural circuitry referred to as the non-homeostatic, or cortico-limbic, system (Kelley et al., 2005; Lutter and Nestler, 2009); this is supported anatomically as many hypothalamic nuclei receive inputs from several relevant cortico-limbic regions (Benarroch, 2010; Berthoud, 2011; Stanley et al., 2011). The non-homeostatic system coordinates metabolic needs with external factors including external challenges, habits, and pleasurable feelings, and enables consumption of palatable foods well beyond the point when energy demands have been met (Kampe et al., 2009; Zheng et al., 2009; La Fleur et al., 2010). The limbic circuitry is known for its involvement in emotion (Davidson and Irwin, 1999), but cortico-limbic circuitry also mediates the rewarding aspects of food, including “liking,” which is the pleasure associated with actual food consumption, and “wanting,” which is the motivation to obtain food (Berridge, 1996).

As stipulated earlier, within the cortico-limbic circuitry are sites involved in feeding, stress and reward. The NAcc appears to be a critical region in feeding, especially of palatable food (Kelley et al., 2005; Alsiö et al., 2010; Miner et al., 2010). Indeed, food reward is capable of eliciting dopamine (DA) release from the NAcc in the same way as do addictive drugs such as cocaine and amphetamine (Hernandez and Hoebel, 1988; Pandit et al., 2011). Parenthetically, stress also elicits DA release from the NAcc (Abercrombie et al., 1989; Deutch and Cameron, 1992; Kalivas and Duffy, 1995), and stress-induced DA release from the NAcc is absent in rats that cannot produce GCs (Rougé-Pont et al., 1998). Importantly, extremely high densities of both BB_1_ and BB_2_ receptors are localized at the NAcc (Ladenheim et al., 1992). In contrast, only low to moderate levels of BB_3_ receptor mRNA are expressed at this site (Zhang et al., 2013).

The amygdala is activated by both pleasant and aversive tastes (O'Doherty et al., 2001b) and contains two nuclei of interest, namely the basolateral amygdala (BLA) and the central amygdala (CeA). Both nuclei are implicated in stress (Davis and Whalen, 2001) as well as reward circuitry (Ahn and Phillips, 2002; Carelli et al., 2003), particularly in the conditioning of reward cues (Mahler and Berridge, 2009; Jones et al., 2010), including food-related cues (Petrovich et al., 2009; Petrovich, 2011). Within the amygdala, a moderate density of BB_1_, BB_2_, and BB_3_ receptor mRNA are expressed at the CeA (Ladenheim et al., 1992), whereas BB_2_ receptor mRNA is highly expressed in the lateral amygdala (part of the BLA complex) (Shumyatsky et al., 2002).

The ACC, which is innervated by the hypothalamic arcuate nucleus via the lateral hypothalamus (Kampe et al., 2009), is involved in emotion (Shackman et al., 2011) as well as higher order processes such as decision-making (Rosenbloom et al., 2012), self-awareness (Allman et al., 2010), attention (Weible, 2013), and reward (O'Doherty et al., 2001a; Berthoud, 2011). Imaging and electrophysiological studies further support involvement of the ACC in food reward as it is responsive to the sensory or hedonic properties as well as the palatability of food (O'Doherty et al., 2001b; Verhagen et al., 2003; Rolls, 2005). We recently showed that activation of GRP receptors in the ACC elicits GRP, but not CRF, release at the BLA, suggesting a functional pathway between these two regions utilizing BBs (Merali et al., 2013). It is of interest to note that there is a population of specialized neurons within the ACC of humans and primates, that selectively express NMB and GRP (Allman et al., 2010); the so called von economo neurons (VENs) are involved in consciously motivated behavior. While they are not as clearly delineated in the brains of lower mammals, NMB and GRP mRNA, are expressed in a restricted population of neurons in the ACC of rodents (Allman et al., 2011) thought to be homologous to VENs in humans and represent an intriguing target for investigation with respect to ingestion-related processes.

## Feeding and stress: overlapping neural modulators

Beyond the overlapping circuitry between stress and feeding, there are also overlapping neurochemical signaling systems. Indeed, it is increasingly being recognized that many of the peptides involved in the regulation of food intake also seem to influence the stress response. Such peptides thus are well positioned to play a role in stress-induced changes in feeding behavior, including stress-induced anorexia or stress-induced obesity. For example, cholecystokinin, a satiety peptide involved in meal termination (Moran, 2006) activates the HPA axis (Antonijevic et al., 2000; Karlsson et al., 2005) and is also a powerful panicogenic agent (Zwanzger et al., 2012). Conversely, the orexigenic peptide neuropeptide Y, suppresses HPA activity and has anxiolytic properties (Antonijevic et al., 2000; Karlsson et al., 2005). In addition, both orexin and ghrelin which promote food intake or leptin which suppresses food intake all stimulate the HPA axis (Ahima and Flier, 2000; Asakawa et al., 2001; Spinazzi et al., 2006; Barson et al., 2013; Uchida et al., 2013). Likewise, BBs appear to have a dual function in feeding and stress responses, which will be further discussed below.

## BBs in feeding and stress: potential role in stress-induced anorexia

BBs influence a wide range of biological processes including thermoregulation, itch sensation, smooth muscle contraction, cell growth, endocrine response as well as numerous behavioral effects (Schjoldager et al., 1991; Itoh et al., 1995; Shumyatsky et al., 2002; Mountney et al., 2008; Merali et al., 2011, 1999; Su and Ko, 2011; Saito et al., 2013). However, this family of peptides, which is distributed throughout the gastrointestinal tract and brain, are widely recognized for their ability to influence digestion and food intake. As the name of one mammalian form, GRP, implies, BBs dose dependently stimulate gastrin and gastric acid secretion when administered peripherally (Knigge et al., 1984; Hildebrand et al., 2001), however, when injected into the brain, BB and GRP are potent inhibitors of gastric acid secretion (Martinez and Taché, 2000). On a behavioral level, both systemic and central administration of BBs suppress food intake and evoke behavioral and physiological responses akin to spontaneous satiety (Kulkosky et al., 1982; Smith and Gibbs, 1984; Merali et al., 1999). Bombesin is the most potent at suppressing food intake (due to activation of both BB_1_ and BB_2_ receptors), followed by GRP and then NMB (Sayegh, 2013). The satiety effects of exogenously administered BBs will not be outlined in further detail as they have been well described in several review papers (Gibbs and Smith, 1988; Merali et al., 1999; Yamada et al., 2002; Majumdar and Weber, 2011; Sayegh, 2013). Additional evidence for a role of BBs in the regulation of food intake comes from studies showing changes in peptide levels or mRNA expression in different metabolic states. For example, our lab has shown changes in tissue levels of immunoreactive (ir)-BBs at specific gut and brain regions in response to food ingestion and deprivation (Merali and Kateb, 1993; Plamondon and Merali, 1997). During a spontaneous meal ingestion, levels of ir-BBs increased significantly at hypothalamic structures including the PVN, arcuate nucleus and dorsomedial nucleus (Plamondon and Merali, 1997). Moreover, interstitial levels of BBs (assessed using push-pull perfusion) at the PVN were higher before meal ingestion and after the meal, as compared to those noted during food ingestion (Plamondon and Merali, 1994). More recently it was shown that food deprivation decreased GRP mRNA expression at the PVN, while a melanocortin agonist increased GRP mRNA at this site (Ladenheim et al., 2009).

The use of knockout strategy has further revealed that a lack of BB_3_ receptors results in hyperphagia, leptin and insulin resistance, glucose metabolism dysregulation and the development of late onset obesity (Ohki-Hamazaki et al., 1997). Moreover, treatment with a novel synthetic BB_3_ agonist results in weight loss and increased metabolic rate in mice and dogs (Guan et al., 2011), supporting evaluation of the BB_3_ receptor as a potential therapeutic target for obesity (Zhang et al., 2013). It is also noteworthy that mice lacking BB_2_ receptors eat more food during a meal than wild type mice and gain more weight over the long term, consistent with a role for this receptor subtype in satiety (Ohki-Hamazaki et al., 1997; Ladenheim et al., 2002). In contrast, mice lacking the BB_1_ receptor showed no alterations in food intake or body weight gain (Ohki-Hamazaki et al., 1999), however, human genetic studies support a strong association between polymorphisms on the NMB gene and increased adiposity and obesity (Bouchard et al., 2004; Spálová et al., 2008; Pigeyre et al., 2010). Interestingly, in adolescence, the association between the polymorphism on the NMB gene and obesity was exacerbated in families of lower socio-economic status (Pigeyre et al., 2010). High fat diets, low physical activity and exposure to chronic stress are more prevalent in families of low socio-economic status (James et al., 1997; Baum et al., 1999).

BBs are also implicated in the mediation of the stress response. BBs are located in all major nodes of the HPA axis including the PVN, the anterior pituitary and the adrenal gland in addition to other stress responsive regions (Merali et al., 2002). Central administration of BBs activates both the HPA axis and the sympathetic branch of the autonomic nervous system as reflected by increased release of ACTH, corticosterone, norepinephrine and epinephrine; these effects are blocked by pretreatment with competitive and specific BB receptor antagonists (Brown et al., 1979, 1988; Gunion et al., 1989; Carver-Moore et al., 1991; Olsen et al., 1992; Malendowicz and Nussdorfer, 1995; Okuma et al., 1996; Au et al., 1997; Garrido et al., 1998, 1999; Malendowicz, 1998).

Considerable evidence suggests that BBs exert some of these effects via activation of CRF neurons. For example, pretreatment with a CRF receptor antagonist can block the endocrine, sympathetic and behavioral effects of central GRP administration (Garrido et al., 1998, 2002; Kent et al., 2001b). Moreover, we reported that central BB administration stimulates the release of CRF from the median eminence (the primary source of CRF release during HPA activation) translating into an increased availability of this peptide downstream at the anterior pituitary (Kent et al., 2001a). Interestingly, there is also recent evidence of co-localization of BB_3_ and CRF receptors within the hypothalamus, including at the PVN and dorsomedial nucleus, yet the functional significance of this overlapping circuitry remains to be determined (Zhang et al., 2013).

Moreover, the BB systems are stress responsive as site-specific alterations in the endogenous levels of BBs and BB receptor densities are observed in response to acute stressor (restraint) exposure, including increased BBs at the hypothalamus and increased BB receptors at the PVN (Kent et al., 1998). Finally, we observed that acute restraint elicits the release of both CRF and BBs at the CeA (Merali et al., 1998), whereas chronic restraint exposure is associated with elevated interstitial levels of GRP at the anterior pituitary (Merali et al., 2009).

**Figure 1:**
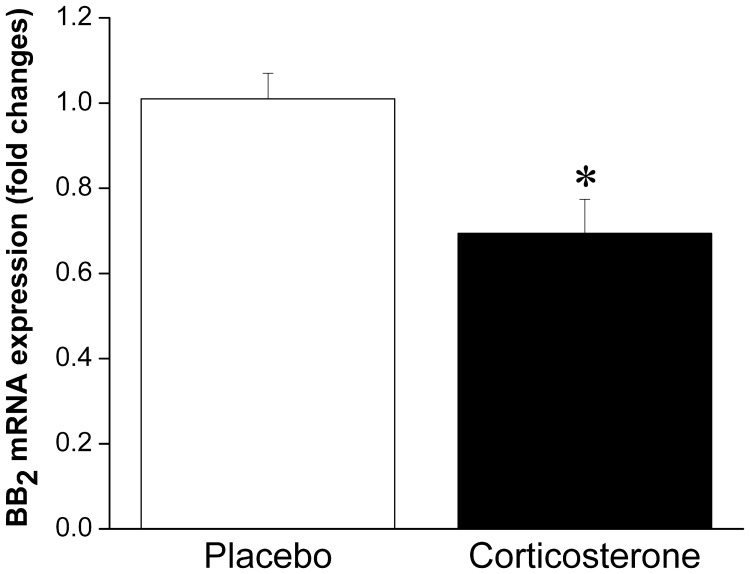
**Mean ± SEM (fold change) of mRNA expression of BB_2_ at the PVN in placebo or corticosterone pellet (100 mg, 21 day slow release) implanted rats.** Chronic corticosterone exposure resulted in a significant reduction in BB_2_ mRNA expression at the PVN. ^*^Significantly different from placebo at *p* < 0.05.

Taken together, these results suggest that under normal, non-stressful conditions, BBs have satiety effects. Given that BBs are released in response to stressor exposure (both acute and chronic), this peptidergic system is likely involved in stress-induced anorexia. Logically, then, weight gain associated with some models of stress (i.e., chronic psychosocial stress) may be linked to a decreased ability to respond to BBs' satiety effects, which is supported by the finding that obese women are less sensitive to BB-induced satiety than lean women (Lieverse et al., 1998), and this is also consistent with the obesity seen in BB receptor knockout mice. We could speculate that reduced sensitivity to BB may be the result of stressor-induced alterations in BB signaling leading to down-regulation of BB receptors at feeding relevant sites, attributable to prolonged release and exposure to BBs. Indeed, we have observed enhanced interstitial level of BBs at the anterior pituitary (located downstream of the hypothalamus) in response to chronic stressor (14 once daily restraint sessions) exposure (Merali et al., 2009), which could provide a mechanism for a stress-induced down-regulation of BB receptors. In support of this contention, we recently observed reduced mRNA expression of BB_2_ receptors at the PVN following chronic corticosterone exposure (unpublished finding; see Figure 1). Moreover, sustained BB exposure (via chronic infusion) resulted in a down-regulation of BB receptors at the PVN and a tolerance development to the feeding suppressant effects of BB (Plamondon et al., 1998).

## BBs in incentive salience and reward processes: potential role in stress-induced obesity

**Figure 2:**
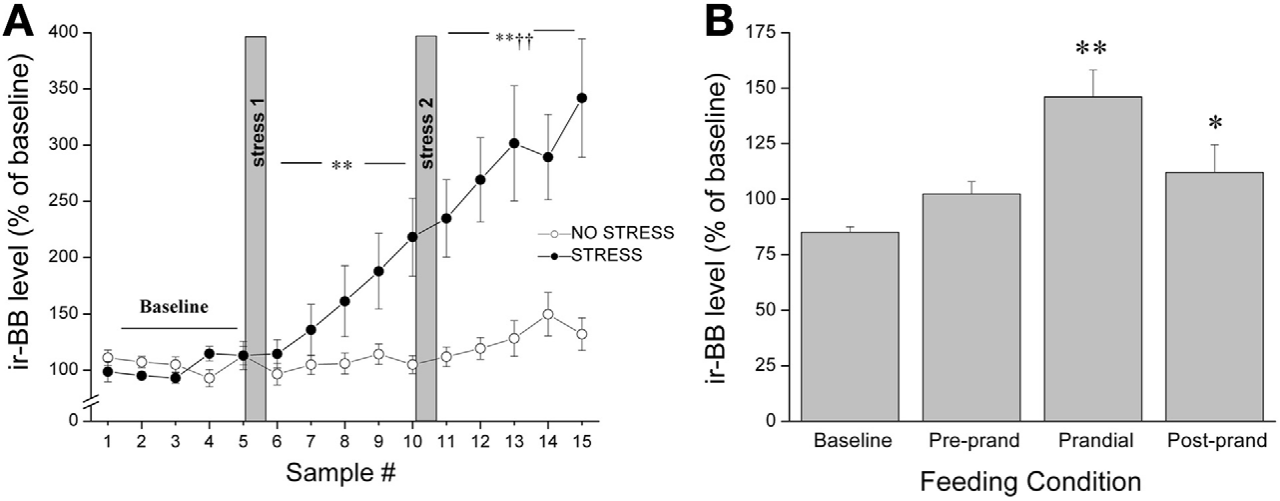
**Release of BBs at the CeA as measured by *in vivo* microdialysis in response to (A) restraint stress exposure (20 min hand restraint on two separate occasions called stress 1 and stress 2) or (B) presentation and ingestion of a palatable snack where microdialysis samples were collected continually and pooled every 30 min for 5 h.** The quantity of food ingested during the 30 min bin was noted and the 30 min period before meal initiation was considered the preprandial period, and the 30 min sample preceding this was considered the baseline. Both stressor exposure and ingestion of a palatable snack elicited a significant increase in the release of BBs (and CRF) at the CeA accompanied by a parallel increase in plasma corticosterone levels. ^*,**^Significantly different from baseline at *p* < 0.05 and *p* < 0.01, respectively. ^††^Significantly different from stress 1 at *p* < 0.01.

It was originally thought that the classic feeding regulators acted predominantly on homeostatic systems to control energy balance; however, increasing evidence suggests that food intake is a much more complex process, involving a much broader array of functions. In keeping with the contention of dual roles in feeding and stress, several years ago we reported that both appetitive (palatable food; graham crackers) and aversive (restraint stress) stimuli provoked *in vivo* release of BBs and CRF at the CeA, with a parallel rise in circulating corticosterone levels (Merali et al., 1998) (see Figure 2). To explain similar neurochemical responses to both aversive and appetitive stimuli, we suggested that rather than evoking fear and anxiety, these so-called “satiety/stress peptides” may serve to draw attention to biologically significant events (or cues) such as those associated with food availability as well as those posing physical threat. This would be akin to dopaminergic responses that might act in a similar capacity (Richardson and Gratton, 1996; Wickelgren, 1997). Indeed, dopaminergic neurons within the prefrontal cortex and NAcc, once thought to be exclusively involved in reward, were subsequently found to be responsive to stressors or stimuli with a negative valence (Horvitz, 2000). These observations led to the suggestions that dopaminergic signals contribute to specific cognitive functions and/or arousal (Richardson and Gratton, 1996; Horvitz, 2000). With time, this idea of stressor-induced increased incentive salience became a cornerstone to Dallman's “comfort food theory of obesity”(Dallman et al., 2005; Dallman, 2010). Her work showed that chronic stressor exposure elicits high levels of GCs and increases synthesis of CRF at stress/reward-responsive cortico-limbic sites like the CeA, which in turn enhances both the drive to consume as well as the salience of palatable foods (Foster et al., 2009). Once consumed, comfort foods themselves activate reward centers to subsequently reduce HPA activity.

Like BBs, the CRF family of peptides potently suppress food intake, and have been implicated in stressor-induced decreases in food intake (Dunn and Berridge, 1990; Koob and Heinrichs, 1999). However, beyond satiety effects, CRF has also been implicated in reward pathways and incentive salience. For instance, increase in the release of CRF or its mRNA expression is provoked by natural rewards and incentive cues, at relevant cortico-limbic sites including the ACC and CeA (Merali et al., 2004; Foster et al., 2009). Moreover, CRF at the NAcc amplifies positive motivation for cued rewards by magnifying incentive salience (Peciña et al., 2006) and injection of CRF at this site elicits conditioned place preference for the chamber paired with CRF; the conditioning is dependent on CRF-induced DA release in this region (Lemos et al., 2012). Finally, in humans, low dose CRF administration increased palatable food consumption in a cortisol dependent manner (George et al., 2010).

**Figure 3:**
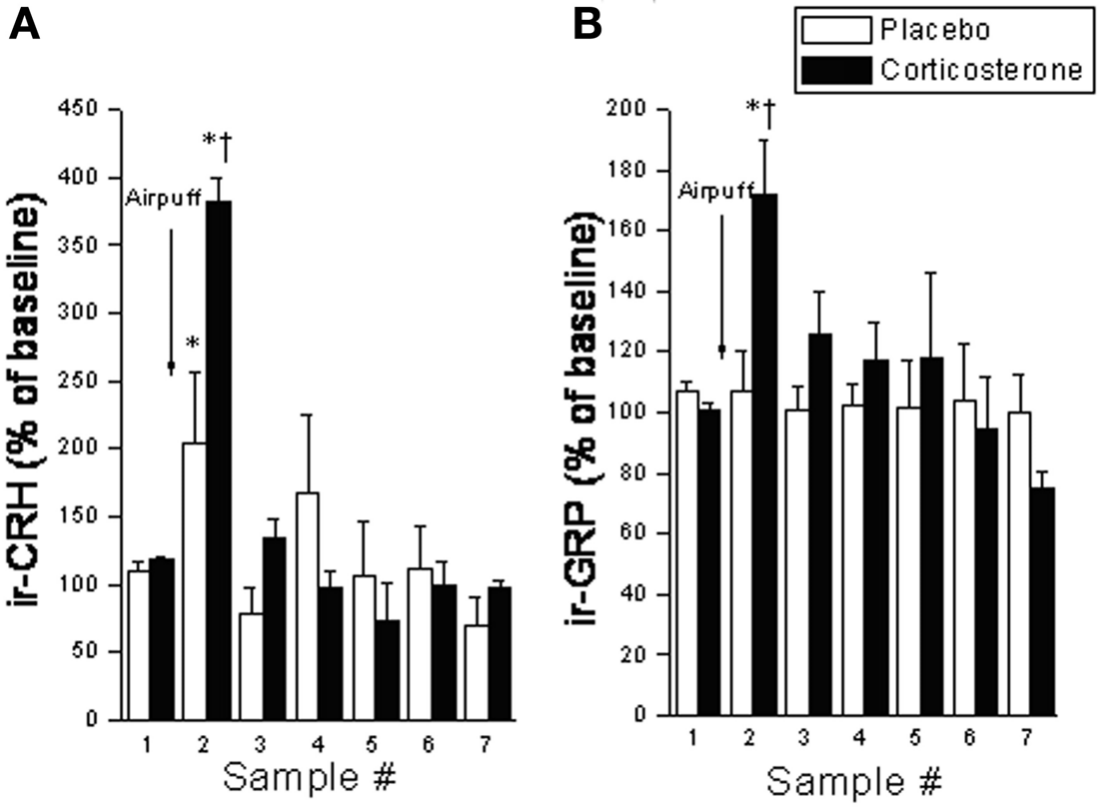
**Interstitial levels of (A) immunoreactive (ir) -CRF or (B) ir-GRP (expressed as a percentage of baseline values) at the CeA under basal conditions and following exposure to an airpuff stressor (5 airpuffs; 5 s puff/min) in placebo or corticosterone pellet (100 mg, 21 day slow release) implanted rats.** Chronic corticosterone exposure potentiated the stressor-elicited release of CRF and GRP. ^*^Significantly different from respective baseline sample at *p* < 0.05. ^†^Significantly different from (sample-matched) placebo control condition at *p* < 0.05.

Despite the provocative data, BBs involvement in incentive salience and reward has yet to be fully investigated. Given the similarities between BBs and CRF, and the fact that many of BBs stress effects appear to be mediated by CRF (as described above), we expect that BBs may similarly affect reward processes. As mentioned earlier, like CRF, BBs are released at the CeA in response to both stressor exposure and ingestion of a palatable snack (Merali et al., 1998). Notably, rats exposed to high chronic (14 days) doses of corticosterone (via systemic pump implants) show exaggerated stressor-provoked GRP (and CRF) release at the CeA and medial prefrontal cortex (which encompasses the ACC) (Merali et al., 2008) (see Figure 3). These findings are consistent with the notion of a sensitized BB release at cortico-limbic structures under stressful conditions (characterized by high GC levels).

In further support of the contended role of BBs' in motivation/reward, these peptides appear to interact with DA at key reward structures. DA is the neurotransmitter most closely associated with reward processes (Wise, 2004, 2006; Covey and Howard, 2011; Volkow et al., 2011), and has been implicated as one of the mediators of food reward processes (Wise et al., 1978; Bassareo and Di Chiara, 1999; Volkow et al., 2011). Our lab has shown robust meal-related fluctuations in levels of BBs at the NAcc (Plamondon and Merali, 1997). Microinjection of BBs at the NAcc elicits a marked increase in locomotor activity that is blocked by pretreatment with a D1 receptor antagonist, implicating BBs' capacity to modulate dopaminergic activity within this structure (Schulz et al., 1984; Johnston and Merali, 1988). Blockade of D1 and/or D2 receptors also attenuates the central BB-elicited increase in locomotor activity and grooming (Piggins and Merali, 1989; Merali and Piggins, 1990). Additionally, BBs increased DA synthesis in the dorsal striatum, olfactory tubercles, and hypothalamus (Widerlöv et al., 1984) and increased the activity of tuberoinfundibular and tuberohypophysial DA neurons (Manzanares et al., 1991).

It is also noteworthy that BBs interact with the inhibitory neurotransmitter GABA. The GABAergic system is critical for the regulation of both reward (Wirtshafter and Stratford, 2010; Welberg, 2012), and stress (Herman et al., 2004), and is thought to be tied to motivational aspects of feeding (Truong et al., 2002; Takagi et al., 2003). Injection of GABA_A_ or GABA_B_ agonists at the NAcc shell produces profound hyperphagia in satiated rats (Stratford and Kelley, 1997; Basso and Kelley, 1999; Baldo et al., 2005). Moreover, chronic stress increased expression of the GABA-producing enzyme glutamic acid decarboxylase (GAD65) at the anterior hypothalamus but decreased GAD65 expression at the dorsal hypothalamus, effects reversed by palatable diet (sucrose) consumption (Christiansen et al., 2011b). GABA is also a key signal in reward pathways, as both a GABA_A_ agonist and amphetamine injected at the NAcc shell increase the breaking point of lever pressing for food reward (Wirtshafter and Stratford, 2010), suggesting the rodents will “work harder” to attain reward. GRP infusion increases GABA efflux at the ventral hippocampus, an effect blocked by a BB_2_ receptor antagonist (Andrews et al., 2000). Moreover, GABAergic interneurons at the lateral amygdala abundantly express BB_2_ receptors (Shumyatsky et al., 2002), and application of GRP stimulates these interneurons to enhance inhibition of principal neurons (Cao et al., 2010). GRP application also facilitates GABA release at the ACC and amygdala (Cao et al., 2010). While the relationship between BBs and GABA at the amygdala is thought to modulate learned fear (Shumyatsky et al., 2002), their interactions in reward pathways, potentially at the NAcc, have yet to be fully elucidated and present an intriguing avenue for further investigation.

Taken together, BBs act at reward sites, potentially through modulation of DA and/or GABA functioning. As such, the necessary “hardware” is available to support a role for BBs in reward processes. Additionally, our lab now has preliminary evidence indicating that microinjection of BBs at the NAcc elicits DA release at this site. Moreover, like for CRF, injection of BBs at the NAcc is capable of eliciting conditioned place preference (manuscript in preparation). Thus, we hypothesize that, like CRF, BBs may act to increase the incentive salience associated with food reward. It has been suggested that the combination of GCs and CRF may act to associate the feeling of stress with the relief of stress by palatable food (Dallman, 2010). Similarly, BBs' ability to increase incentive salience, through GCs, and/or through interactions with DA or GABA, could strengthen a learned association between stress and palatable food, to enhance the rewarding properties of palatable food and to promote palatable food consumption.

In the realm of addiction research, which may also apply to excessive palatable food intake, an alternate, more common view for CRF, is that rather than increasing incentive salience or reward, it actually decreases reward (increases the threshold for reward). Indeed, behavioral consequences of stressor exposure and CRF release are typically characterized by increased anxiety and anhedonia (Cottone et al., 2009; Koob, 2013). Therefore, increased intake of drugs of abuse or palatable food during stress may provide a means to counteract the negative aversive (allostatic) state (Cottone et al., 2009; George et al., 2012; Koob, 2013). Likewise it may be argued that under stress, BBs increase the “hedonic threshold” resulting in the need for increased palatable food consumption to achieve reward. While this remains a possibility, the ability of BBs (and CRF) to increase conditioned place preference when injected into the NAcc cannot be explained within this framework. Also inconsistent with this theory, is the ability of BBs to improve emotional states. For example, central administration of GRP (injected i.c.v or localized at the CeA, BLA or ACC), attenuated the fear potentiated startle (FPS) response as well as the expression of learned fear (as seen by reduced levels of freezing) in response to contextual cues (i.e., in the context in which animals had previously been exposed to shock), and to a tone that had previously been paired with a shock (Mountney et al., 2006, 2008; Merali et al., 2011). Moreover, mice lacking BB_2_ receptors exhibit depressive-like behaviors (Monje et al., 2011).

Overall, therefore, based on evidence presented, we maintain the hypothesis that activation of BBs within cortico-limbic circuitry may, under certain circumstances, increase incentive salience/reward which may ultimately lead to weight gain/obesity. In suggesting this hypothesis, it is recognized that at first blush, it appears incompatible with the observed link between BB receptor knockout models (BB_2_ and BB_3_) and eventual weight gain/obesity. However, it should be emphasized that BB receptor knockout strategy impacts all receptors and related circuitry. In the case of CRF, research has shown that whereas the impact of chronic stressor exposure (or chronic GC exposure) predictably down-regulates hypothalamic CRF (particularly at the PVN), it “paradoxically” up-regulates or sensitizes the CRF system at cortico-limbic sites such as the CeA (Swanson and Simmons, 1989; Makino et al., 1994, 1999; Cook, 2002). Likewise it is possible that the food/stress elicited changes in specific circuits endowed with BB receptors may respond differentially; an effect not functionally captured through knockout strategy.

## Conclusions

In sum, we propose a dual function for BBs in stress and feeding. Most obvious is a role for BBs in stress-induced anorexia. Exogenous administration of BBs potently suppress food intake and BBs are released centrally in response to stressor exposure (both acute and chronic). Weight gain associated with some models of chronic stress could be linked to an inability to respond to satiety signals, including those of BBs. As previously alluded, there is evidence of BB receptor down-regulation (at feeding relevant brain sites) following prolonged corticosterone exposure or chronic BB administration (Plamondon et al., 1998) which could be a mechanism for reduced sensitivity to the satiety effects of BBs (disinhibition).

While increased BB signaling within feeding relevant homeostatic circuitry may contribute to stress-induce anorexia or conversely, an impairment of BB signaling within this same circuitry may promote stress-induced obesity (under some circumstances), we further propose that *increased* BB signaling within cortico-limbic circuitry may also contribute to stress-induced obesity. It is when stressor exposure is combined with a palatable food diet, that we believe this second scenario becomes relevant. BBs are uniquely distributed within key cortico-limbic brain regions linked to reward; most notably at the Nacc, ACC, and amygdala. Moreover, we now have direct evidence that BBs, at the NAcc, induce conditioned place preference which strongly supports their involvement in reward-mediated processes. It is our contention that release of BBs at these cortico-limbic structures may serve to increase incentive salience and/or reward associated with palatable food; indeed future studies directly linking BB-induced reward/incentive salience with increased palatable food consumption need to be carried out to fully validate this hypothesis. Through their interaction with GCs, DA and/or GABA, BBs may enhance the rewarding/stress buffering properties of palatable food and/or strengthen a learned association between stress and palatable food, which may in turn further promote palatable food consumption ultimately leading to obesity.

### Conflict of interest statement

The authors declare that the research was conducted in the absence of any commercial or financial relationships that could be construed as a potential conflict of interest.
